# Chimeric Antigen Receptor T Cell Therapy for Hepatocellular Carcinoma: Where Do We Stand?

**DOI:** 10.3390/ijms25052631

**Published:** 2024-02-23

**Authors:** Ioanna Aggeletopoulou, Maria Kalafateli, Christos Triantos

**Affiliations:** 1 Division of Gastroenterology, Department of Internal Medicine, University Hospital of Patras, 26504 Patras, Greece; iaggel@hotmail.com; 2 Department of Gastroenterology, General Hospital of Patras, 26332 Patras, Greece; mariakalaf@hotmail.com

**Keywords:** hepatocellular carcinoma, chimeric antigen receptor, immunotherapy, adoptive T cell therapy, tumor microenvironment, T cell exhaustion, cytotoxicity

## Abstract

Hepatocellular carcinoma (HCC) remains a global health challenge that urgently calls for innovative therapeutic strategies. Chimeric antigen receptor T cell (CAR T) therapy has emerged as a promising avenue for HCC treatment. However, the therapeutic efficacy of CAR T immunotherapy in HCC patients is significantly compromised by some major issues including the immunosuppressive environment within the tumor, antigen heterogeneity, CAR T cell exhaustion, and the advanced risk for on-target/off-tumor toxicity. To overcome these challenges, many ongoing preclinical and clinical trials are underway focusing on the identification of optimal target antigens and the decryption of the immunosuppressive milieu of HCC. Moreover, limited tumor infiltration constitutes a significant obstacle of CAR T cell therapy that should be addressed. The continuous effort to design molecular targets for CAR cells highlights the importance for a more practical approach for CAR-modified cell manufacturing. This review critically examines the current landscape of CAR T cell therapy for HCC, shedding light on the changes in innate and adaptive immune responses in the context of HCC, identifying potential CAR T cell targets, and exploring approaches to overcome inherent challenges. Ongoing advancements in scientific research and convergence of diverse treatment modalities offer the potential to greatly enhance HCC patients’ care in the future.

## 1. Introduction

Hepatocellular carcinoma (HCC) is the most common primary liver cancer (more than 90%), and its incidence can vary depending on the geographical region, ethnicity, genetic background, and other factors [1]. HCC remains a global health care challenge with a million cases predicted to occur each year by 2025 [1]. Currently, HCC ranks as the fifth most prevalent cancer worldwide and it has been associated with a five-year survival rate of approximately 18%, standing as the third-leading cause of cancer-associated mortality [2,3,4]. The pathogenesis of HCC is still vague. Major etiological risk factors for HCC development include viral hepatitis (hepatitis B and hepatitis C), alcoholic liver disease and metabolic dysfunction-associated steatotic liver disease (MASLD). This background becomes more complex when tobacco consumption, excessive aflatoxin accumulation, and even rare monogenic disorders add on as possible pathogenic factors for HCC [5]. HCC is strongly associated with liver cirrhosis in 90% of cases with an annual incidence of 2–4% in this setting [1,3,6]. During the last decade, the management of HCC has significantly progressed, with treatment decisions primarily based on the Barcelona Clinic Liver Cancer (BCLC) staging system which takes into account the tumor stage and the anticipated benefits from each intervention [7,8]. Surgical resection, liver transplantation and local ablation are the most potent therapies for HCC; however, their effectiveness is restricted to patients with an early diagnosis and those with sufficient liver function to tolerate surgery [1,3]. Patients at intermediate stages are candidates for transarterial chemoembolization (TACE) whereas those with advanced disease undergo systemic and immune therapies [1,9,10]. However, despite these therapeutic approaches and their combinations, the 5-year post-treatment recurrence rate in the early HCC stage is very high, approaching 70%, whereas in the advanced stages the median survival probability is very diminished [11]. Immunotherapy aims to activate or enhance the immune system through an improvement of the natural defense mechanisms that recognize, attack, and destroy tumor cells [12]. Immunotherapy can be categorized into two main types based on the specificity of the targeted tumor, i.e., tumor-specific immunotherapy and tumor-nonspecific immunotherapy. Non-specific immunotherapy includes cytokines, cytokine-induced killer cells, and natural killer (NK) cells. On the other hand, tumor-specific immunotherapy aims to enhance the immune system’s ability to recognize and fight against tumor cells, leading to an anti-tumor effect. In the context of tumor-specific immunotherapy, adoptive cell therapy (ACT) involves strategies such as tumor-infiltrating lymphocytes (TIL) or genetically engineered T cells expressing novel T cell receptors (TCR) or chimeric antigen receptors (CAR) [13,14,15].

The challenges associated with the in vitro expansion of tumor-specific T cells boosted the creation of TCR-engineered lymphocytes; nonetheless, these cells have the limitation of recognizing only the tumor antigens that are presented by the major histocompatibility complex (MHC), also known as human leukocyte antigen (HLA) in humans [16]. On the other hand, the introduction of synthetic CARs allows circumvention of MHC restriction, enabling targeted cytotoxicity against a surface molecule on malignant cells [17]. T cells isolated from a patient (or an allogeneic donor) undergo genetic modification to express CARs, followed by expansion and subsequent infusion into this patient. This approach addresses the fact that tumor cells frequently downregulate MHC molecules, rendering them unable to present antigens to conventional T cells.

This review aims to provide a comprehensive exploration of the adoptive immunotherapy for HCC, focusing on CAR T cells efficacy against tumors and the biological rationale behind this process. In parallel, the most recent therapeutic advancements in the HCC setting will be discussed. Special attention will be given on CAR T cell targets for HCC and on promising strategies to ameliorate CAR-mediated T cell efficacy.

## 2. CAR T Cell Structure

**Figure 1:**
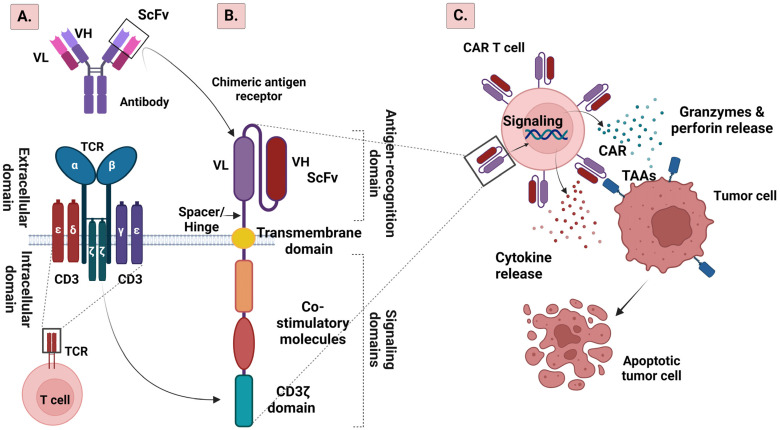
(**A**). Schematic illustration of T cell receptor (TCR) and (**B**). chimeric antigen receptor (CAR) structures. (**C**). Mechanism of CAR T cell treatment. CAR T cell employs the single-chain variable fragment (scFv) domain to identify and attach to the tumor-associated antigen (TAA) located on the surface of the tumor cell. This interaction triggers signaling in CAR T cell through the CD3ζ endodomain module, initiating cytotoxic functions that involve the generation of perforins, granzymes, and cytokines. Created with BioRender.com (accessed on 5 January 2024). Abbreviations: VL, variable fragment light chain; VH, variable fragment heavy chain; scFv, single-chain variable fragment; TCR, T cell receptor; CAR, chimeric antigen receptor; TAA, tumor-associated antigen.

TCR is located on the surface of a T cell enabling recognition and binding to specific antigens (Figure 1).

In CAR T cell therapy, the TCR is often modified or replaced with a synthetic receptor. The structure of a CAR T cell consists of four main functional domains: (a) the extracellular structural domain, which is responsible for identification and attachment to antigens, (b) the hinge region, (c) the transmembrane structural domain, which induces the functionality and augments the expression of the CAR, and (d) the intracellular structural domain, which is responsible for the co-stimulation and transmission of signals [18,19] (Figure 1). A crucial component of the extracellular structural domain is the single-chain variable fragment (scFv) which serves as the antigen recognition domain [20]. It typically derives from a tumor antigen-reactive antibody and is specific to the target antigen expressed on cancer cells, thus avoiding the restriction of MHC-peptide presentation. ScFv is a chimeric protein composed of a light-chain and a heavy-chain antibody variable domain [20]. The hinge region (or spacer) connects the scFv protein to the transmembrane domain of the CAR [21]. It provides flexibility and allows the scFv to both move freely and bind to target antigens [18]. The transmembrane domain anchors CAR to the T cell membrane and helps with signal transduction [18,22]. The intracellular signaling domains (or endodomains) are critical for the transmission of activation signals to the T cell when the scFv binds to the target antigen [18]. There are typically two signaling domains in a CAR T cell, namely the CD3ζ (CD3 zeta) and the co-stimulatory domain (Figure 1). The CD3ζ domain derives from the TCR complex and is responsible for T cell activation [23]. In addition to CD3ζ, many CAR T cells incorporate a co-stimulatory domain, such as CD28, 4-1BB, CD27 or CD134. Co-stimulatory domains enhance the persistence and efficacy of CAR T cells by providing additional activation signals and promoting T cell proliferation [24].

**Figure 2:**
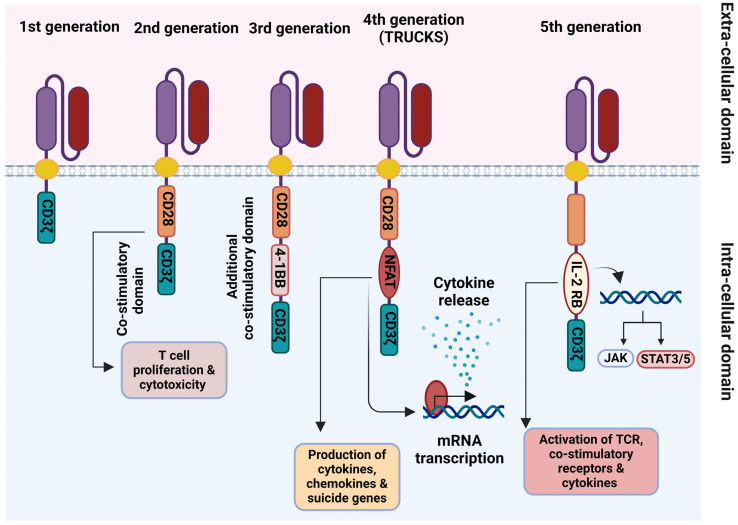
Schematic representation of the 1st–5th generations of chimeric antigen receptors (CARs). Created with BioRender.com (accessed on 5 January 2024). Abbreviations: TRUCKs, T cells redirected for universal cytokine-mediated killing; NFAT, nuclear factor of activated T cells; IL2RB, interleukin 2 receptor subunit beta; TCR, T cell receptor.

The progress in CAR development over the past thirty years can be broadly categorized into five generations (Figure 2) based on the structure and composition of the intracellular signaling domain, with the fifth generation of CARs to be under active development.

### 2.1. From the First to the Fifth Generation of CAR T Cells

The first-generation of CARs is structured as an antigen-binding domain (scFv) linked to a single intracellular signaling domain, typically CD3ζ. These CARs enable basic T cell activation upon antigen recognition but exhibit limited persistence and efficacy due to the absence of co-stimulatory and cytokine signaling [25,26]. Moving to the second-generation of CARs, the design incorporates the components of the first-generation but adds one or more co-stimulatory domains, such as CD28 or 4-1BB (CD137). This modification results in improved T cell activation, proliferation, and persistence leading to enhanced antitumor activity [27,28]. The third generation takes a step further with the inclusion of multiple co-stimulatory domains alongside the CD3ζ signaling domain, creating a tripartite structure. This evolution further enhances T cell activation, cytokine production, and proliferation with even more improved anti-tumor responses [25]. In the fourth generation of CARs, the structure builds upon the third generation by incorporating an additional cytokine or immune modulator transgene, which is expressed either constitutively or inducibly upon CAR activation. This addition is designed to modulate the local tumor microenvironment with the secretion of cytokines, thereby enhancing T cell function and recruiting other immune cells. T cells transduced with this kind of CARs are called T cells redirected for universal cytokine-mediated killing (TRUCKs) [29]. The fifth generation of CARs introduces additional genetic modifications to CAR T cells providing them the advantage to express synthetic receptors that respond to specific signals in the tumor microenvironment. Thus, this generation of CARs grants a more adaptable and controlled approach, allowing for the fine-tuning of CAR T cell responses based on signals received from the tumor site [30].

Recent developments in CAR T cell technology go beyond traditional CARs, introducing advanced systems known as Boolean logic-gated CAR T cells. These innovations aim to enhance the specificity of CAR T cells by providing better control over their activities and addressing limitations associated with conventional CARs [31,32]. These cutting-edge CAR technologies are designed to increase the cancer-specificity of CAR T cells, ultimately improving the treatment effectiveness while minimizing adverse toxicities. Various forms of logic gating are employed, with AND-, OR-, NOT, and IF-Better logic gates being among the most common options [32,33].

### 2.2. AND Logic Gate CAR

AND logic gate CAR, termed also as split-recognition CAR, involves the separation of activation signals, with one receptor incorporating the primary activation signal (CD3ζ), and the other construct containing co-stimulatory domains like CD28 and 4-1BB. AND gate CAR systems become operational only in the presence of two antigens on a cancer cell. In this system, T cells are crafted with a CD3ζ CAR specific to one antigen and a co-stimulatory receptor (CCR) directed toward a second antigen [34,35].

### 2.3. OR Logic Gate

In this logic gate, multiple potential input signals exist, and any of them can lead to the desired outcome. This represents a multi-antigen approach where recognition of one or more targeted CAR T cell antigens is required. Multi-antigen CAR T cells are created using methods like bicistronic CAR, loopCAR or, tandem (tan) CAR, introducing two different CARs into the same T cell. This is distinct from monovalent conventional CAR T cells, which target only a single antigen [31]. This approach is beneficial in tackling the challenge of antigen escape associated with tumors. Bicistronic or dual CARs transduce two CAR constructs into the same T cell, enabling them to target multiple tumor-related antigens (TAAs). CAR T cells may be genetically modified to express CARs directed at three or more antigens, referred to as triCARs or quad-CARs. Conversely, tanCAR consists of two distinct scFv binding domains joined in tandem within a single CAR, targeting two different TAAs. This configuration demonstrates synergistic antitumor activity when both antigens are recognized simultaneously. Additionally, loopCAR is a recent CAR characterized by a looped structure of tanCAR constructs [36].

### 2.4. NOT Logic Gate

The NOT logic gate, also called inhibitory CARs (iCARs), is designed to include inhibitory signaling instead of activation domains as the internal signaling component of the off-target CAR construct. Inhibitory signals are intended to prevent the activation of the CAR T cell when exposed to an off-target antigen [37]. The NOT logic gate recognizes antigens present on normal tissue but absent on tumor tissue and is linked to the signaling domain of a suppressive co-receptor. iCAR is co-expressed with CARs that precisely target the antigen of interest, mitigating the risk for autoreactivity to bystander tissues. This represents a crucial strategy to avert the occurrence of on-target, off-tumor toxicity.

### 2.5. IF-Better Gate CAR

IF-Better gate CAR is an innovative CAR construct that permits killing solely in response to high CAR target expression but not when low unless a CCR target is also present. The interaction of CCR with the target antigen enhances co-stimulation and avidity, thus enabling increased CAR sensitivity intentionally limited to target cells which express both antigens [38].

### 2.6. SUPRA CAR System

The SUPRA CAR system is a CAR system consisting of a soluble antigen-binding domain (zipFv) and a universal signal transduction receptor (zipCAR) expressed on T cells, aiming to enhance specificity and controllability [39]. The zipFv incorporates a scFv and a leucine zipper, while the zipCAR includes intracellular signaling domains and an extracellular cognate zipper that specifically binds to the zipper on the zipFv. These zippers facilitate the binding between the target antigen and the zipCAR-expressing T cells, inducing T cell responses. This CAR design responds to combinatorial antigens in target cells, allowing for ON/OFF switching to finely tune T cell activation and to implement AND logic gating. The SUPRA CAR was further developed to incorporate a separate suppressive domain, adding NOT logic to its capabilities [40].

## 3. CAR T Cell Manufacturing Process and Challenges

The CAR T cell manufacturing process involves leukapheresis to collect cells, followed by elutriation for myeloid cell removal, T lymphocyte enrichment, transgene delivery with a viral vector, and ex vivo T cell expansion [41]. A key challenge in cellular manufacturing is the effective isolation of T cells from heterogeneous leukapheresis samples, containing various cell types like T cells, myeloid cells, NK cells, erythroid cells, and malignant cells [41]. Contaminants, especially inhibitory cell types, can impact CAR T cell expansion, both in vitro and in vivo. Further challenges include the contamination of autologous peripheral blood mononuclear cells with monocytes, which lead to poor outcomes [42]. Moreover, in one rare case, CAR was inadvertently transduced in a leukemia cell during T cell manufacturing. This resulted in CAR binding in cis to the CD19 epitope on the surface of a leukemia clone that had undergone extensive expansion in a patient with acute lymphoblastic leukemia, concealing the clone from detection by anti-CD19 CAR T cells [43].

To ensure clinical benefits from cell-based gene therapies, sustained transgene expression is often essential. Murine gammaretroviruses and lentiviruses, two clinical gene therapy vector systems, provide prolonged CAR transgene expression [41]. Clinical evidence supports the safety of retroviral vectors in human T cells with prolonged CAR T cell persistence in patients treated for human immunodeficiency virus (HIV) infection [44]. However, retroviral vectors pose higher risks when utilized in human stem cells [45]. Conversely, lentiviral vectors, particularly third-generation self-inactivating ones, have a lower risk of insertional mutagenesis and exhibit greater efficacy in genetically engineered human T cells [46,47].

T helper (CD4+) and T cytotoxic (CD8+) cell subset compositions in cancer patients differ from those in healthy individuals, with potential influences on the effectiveness of CAR T cell products. Stem cell-like memory T cells (Tscm), identified by Gattinoni et al., possess self-renewing and multipotent characteristics, demonstrating superior anti-tumor activity when enriched during CAR T cell culture [48]. Manipulating CAR T cells to have pre-defined CD4+:CD8+ compositions or infusing a defined ratio of early memory CD8+ and CD4+ T cells may enhance efficacy [49,50]. Furthermore, considering that the presence of antigen-experienced T cells can hinder the anti-tumor function of less differentiated lymphocytes [50], adopting strategies to enrich early memory subsets at the initiation of CAR T cell manufacturing or during culture could enhance the efficacy of the engineered cellular product. In parallel, strategies like shortening culture duration [51], preventing telomere loss [52], changing metabolic programming [53], utilizing homeostatic cytokines [54] and modifying epigenetic modulation [55,56] can potentially enhance CAR T cell potency.

## 4. The Role of Tertiary Lymphoid Structures (TLSs) in CAR T Therapy

TLSs are specialized, ectopic lymphoid-like formations that emerge at sites of prolonged inflammation or chronic immune activation, distinct from conventional lymphoid organs like lymph nodes or the spleen. These structures share the features of secondary lymphoid organs and develop in non-lymphoid tissues in response to persistent antigenic stimulation, often seen in chronic infections, autoimmune disorders, transplanted organs, or cancer [57,58,59,60,61,62]. Recent findings underscore the significance of TLSs in orchestrating immune responses against tumors [63].

TLSs exhibit a degree of structural organization with discernible zones akin to secondary lymphoid organs [64]. These zones typically comprise areas enriched in T cells, B cells, dendritic cells (DCs), follicular dendritic cells (FDCs), and high endothelial venules (HEVs) [64]. TLSs can influence local immune responses by promoting the recruitment and activation of immune cells, contributing to surveillance against tumor cells, shaping the dynamics of the tumor microenvironment and impacting overall anti-tumor immunity [62,65]. TLSs can be found in several solid tumors including HCC [66]. The prominent advantages offered by TLSs have guided research focus on probable therapeutic applications; the presence of TLSs is being explored both as a potential biomarker and a therapeutic target either as an independent treatment modality or as a complement in adoptive transfer-based cell therapies, such as CAR T cell therapy [65,67,68,69,70].

The presence of TLSs in the tumor microenvironment may present significant implications for CAR T cell therapy. These structures may influence CAR T cell therapy in several ways; research has verified the role of TLSs in the infiltration of CAR T cells [71]. TLSs are characterized by a rich population of immune cells capable of presenting antigens and by a stable endothelial system; this environment supports the survival of CAR T cells within the tumor. In parallel, TLSs play a role in the promotion of ongoing influx of naive immune cells, contributing to the maintenance of overall immunity. This continuous influx has the potential to significantly enhance the effectiveness of CAR T cells [72]. Through the promotion of TLSs maturation, the infiltration of CAR T cells would be enhanced, thus leading to tumor tissue clearance. Furthermore, CAR T cells, through recognition and engagement with cancer cells, release cytokines that can attract other immune cells [73,74]. This cytokine release may contribute to the formation and maintenance of TLSs within the tumor [75,76].

The pivotal role of antigen-presenting cells in TLS formation and function [77], coupled with the ability of TLSs to recruit effector cells in the tumor microenvironment [78], highlights the development of an artificial or inducible TLS (iTLS) as an adjuvant for therapies involving CAR T or the adoptive transfer of TILs [79].

## 5. The Role of T Cell Depletion in CAR T Cell Therapy

T cell depletion refers to the reduction or removal of T cells from a biological sample, a process which can be performed in vivo, ex vivo, or in vitro [80]. This depletion can be achieved by various mechanisms, such as physical separation methods including centrifugation or filtration, chemical treatments including monoclonal antibodies specific to T cell surface markers or chemotherapeutic agents which induce apoptosis of T cells. Other strategies for T cell depletion may involve the use of immunomagnetic bead-based approaches, photodynamic therapy in which T cells are sensitized to light through the incorporation of photosensitive compounds and, lastly, by in vivo depletion using antibodies against T cells [80,81].

The interest in T cell depletion in cancer has increased alongside the evolving research on immunotherapies, particularly those directed against self-antigens. Snook et al. described the mechanism of antigen-specific CD4+ T cell tolerance, where immunotherapeutic reactions are restricted to the inherent self-antigen guanylyl cyclase c (GUCY2C) in colorectal cancer [82]. Nonetheless, in certain scenarios, the presence of selective CD4+ T cell tolerance offers a distinctive therapeutic avenue to enhance immune and antitumor responses targeting self-antigens. This can be achieved without triggering autoimmunity, through the integration of self-antigen-independent CD4+ T cell epitopes into cancer vaccines [82].

In the context of CAR T therapy, T cell depletion may contribute to the reduction of the graft-versus-host disease (GVHD) risk in allogeneic CAR T therapies [83,84]. This is crucial to ensure that the infused T cells primarily target cancer cells and not normal tissues [83]. Prolonged anti-tumor activity could be achieved with the improvement of CAR T cell persistence. Depletion of endogenous T cells before infusion can create space and resources for the expansion and persistence of CAR T cells in the patient’s body [85]. Paszkiewicz et al. showed that targeting epidermal growth factor receptor with the IgG1 monoclonal antibody cetuximab led to the eradication of CD19 CAR T cells at both the early and late stages following adoptive transfer in mice [85]. This results in the full and durable restoration of functional normal B cells, preventing tumor relapse [85]. Relatively common events during CAR T therapies are neurologic toxicity and the development of cytokine release syndrome (CRS) which is characterized as an excess of immune-regulatory cytokines and factors, as well as immune responses mounted by the recipient’s immune system against the infused CAR T cells [86,87]. T cell depletion can reduce the likelihood of an immune response against the infused CAR T cells, which might otherwise limit their effectiveness or lead to their premature elimination. As far as CRS is concerned, T cell depletion might probably contribute to the management of CRS severity [84,87].

## 6. CAR T Cell Therapy Targets for HCC

**Figure 3:**
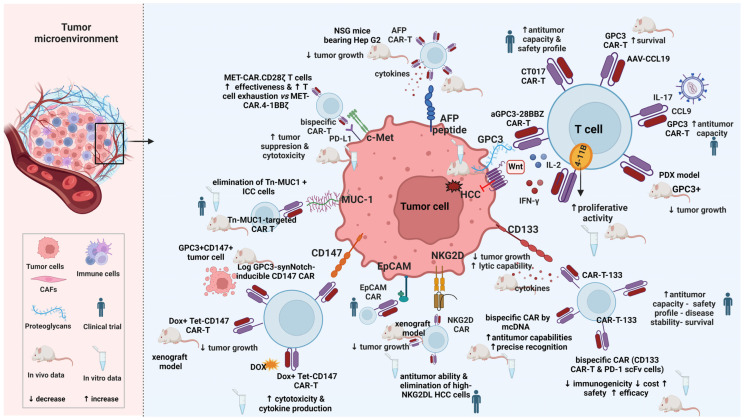
CAR T cell therapy targets for HCC. Created with BioRender.com (accessed on 5 January 2024). Abbreviations: CAF, cancer-associated fibroblasts; CAR, chimeric antigen receptor; MUC1, mucin 1; ICC, intrahepatic cholangiocarcinoma; Dox, doxycycline; EpCAM, epithelial cell adhesion molecule; NKG2D, NK group 2 member D; NKG2DL, NKG2D ligand; HCC, hepatocellular carcinoma; mcDNA, non-viral minicircle DNA; scFv, single-chain variable fragment; GPC3, glypican-3; PDX, patient-derived xenograft; IFN-γ, interferon γ; IL-2, interleukin 2; AAV-CCL19, adeno-associated virus bearing the CCL19 gene; AFP, alpha-fetoprotein.

CAR T cell treatment has shown impressive results in hematological malignancies and the U.S. Food and Drug Administration approved this therapy as gene therapy in this setting [88,89], paving the way for the extension of this approach to solid tumors including HCC. Currently, there has been significant progress in clinical trials and in preclinical animal models utilizing CAR T cells in the setting of HCC (Figure 3).

### 6.1. Glypican-3 (GPC3)

GPC3, a proteoglycan consisting of 580 amino acids with heparan sulfate, is upregulated in various malignancies including HCC [90,91,92]. Conversely, it either lacks or exhibits minimal expression in normal tissues, including normal liver and cirrhotic tissues. The expression of GPC3 has been documented in 72% of HCC patients, and GPC3 serum levels were significantly elevated in 53% of these patients [91]. The proposed role of GPC3 involves the induction of HCC development through stimulation of the Wnt signaling pathway [93]. Silencing GPC3 on HCC cells resulted in impaired proliferation and invasion capacities, indicating the involvement of GPC3 expression in these processes [94]. Furthermore, the influence of serum GPC3 (sGPC3) on CAR T treatment is important as sGPC3 has been associated with poor prognosis in postoperative HCC patients [95]. Sun et al. have shown that sGPC3 can competitively bind to CARs carrying GPC3 on their membrane but is unable to effectively activate CAR T cells [96]. Given its high specificity and sensitivity, GPC3 has been utilized as a target for both HCC diagnosis and treatment.

CAR T cells that target GPC3 have been constructed by Gao et al. [97]. The killing efficacy between first (aGPC3-Z CAR T) and third (aGPC3-28BBZ CAR T) generation GPC3-targeted CAR T therapy was compared and the findings demonstrated that GPC3 CAR T cells effectively halted the development of HCC cells, both in vivo and in vitro, with third-generation GPC3 CAR T cells exhibiting superior effectiveness compared to the first generation ones through higher secretion of both IL-2 and IFN-γ [97]. Li et al. revealed that T cells signaling through CD28 demonstrated higher cytotoxicity in vitro, while CAR T cells containing the 4-1BB co-stimulatory domain displayed enhanced proliferative activity, both in vitro and in vivo [98]. These data suggest that the selection of the co-stimulatory domain could influence the behavior of CAR T cells.

To enhance therapeutic efficacy, CAR T cells expressing IL-7 to stimulate proliferation and CCL19 to boost migration were developed. Research findings demonstrated that the inclusion of IL-7 and CCL19 into CAR T cells significantly enhanced their antitumor capacity. Remarkably, in a phase I clinical trial (NCT03198546), these modified CAR T cells completely eradicated the tumor 30 days after intra-tumor injection in a patient with advanced GPC3+ HCC [99]. Another study confirmed that pretreating the tumor with a recombinant adeno-associated virus bearing the *CCL19* gene (AAV-CCL19) increased the infiltration of GPC3 CAR T cells into the tumor tissue and significantly extended the survival of mice [100]. A phase I trial showed promising antitumor capacity and manageable safety profile of CT017 CAR T cells (cells engineered to co-express GPC3 and RUNX3, a trigger of CD8+ T cell infiltration) in patients with advanced HCC [101].

Double-target CAR T cells against GPC3 and PD-1 were evaluated and the results showed limited PD-1-PD-L1 binding and sustained cytotoxicity to PD-L1+ HCC cells [102]. The low expression of inhibitory receptors in double-target CAR T cells facilitated the suppression of tumor and extended survival in PD-L1+ HCC models compared to their single-target cells [102]. Jiang et al. established three patient-derived xenograft (PDX) models of HCC with GPC3-positive expression [103]. This research validated that GPC3 CAR T cells inhibited tumor growth, albeit with varying effectiveness attributed to the different expression of PD-L1 on tumor cells [103]. This implies that a plausible strategy for achievement of enhanced efficacy in the elimination of PD-L1-positive HCC involves combining CAR T therapy with immune checkpoint inhibitors. Up to date, numerous clinical trials investigating GPC3 CAR T therapy for advanced HCC are underway.

### 6.2. Alpha-Fetoprotein (AFP)

AFP, a secreted glycoprotein, is comprised of 591 amino acids with 4% carbohydrate residues. AFP is implicated in several crucial physiological processes, including transport, immunosuppression and apoptosis [104]. The serum concentration of AFP is over-expressed in conditions such as HCC, hepatoblastoma and teratoma in adults, featuring this protein as a valuable serum marker for tumor diagnosis and drug resistance and for monitoring therapeutic efficacy [105,106]. Taking into consideration that CAR T cells recognize tumor surface antigens but not secreted or intracellular ones, Liu et al. developed AFP CAR T cells capable of selective binding to the AFP_158–166_ peptide which is presented by HLA-A02:01 on the surface of tumor cells in vivo [107]. Subsequently, these CAR T cells degranulated, secreted various cytokines and lysed HLA-A02:01+/AFP+ tumor cells [107]. Furthermore, the administration of AFP-CAR T cells in NSG mice bearing Hep G2 tumors intravenously resulted in swift and significant inhibition of tumor growth [107]. In a pre-existing intraperitoneal liver cancer xenograft model, AFP-CAR T cells exhibited potent antitumor activity [107].

### 6.3. CD133

CD133, a transmembrane glycoprotein, is highly expressed in various cancers including HCC [108]. It has been characterized as a marker for cancer stem cells (CSCs), playing an important role in tumor survival, proliferation, metastasis, and recurrence. CD133 cells are exclusively present in HCC tissues, but not in non-malignant liver tissues, suggesting that CD133 expression in HCC cells may contribute to tumor growth and metastasis potential [109]. Evidence has shown that increased CD133 levels in HCC patients correlate with shorter overall survival and elevated recurrence rates [110]. Thus, CD133 may be considered as a potential molecular target for immunotherapy in patients with CD133+ HCC. CD133-specific CAR-modified T cells (CAR T-133) were generated and their use demonstrated impressive lytic capabilities and high production of cytokines against CD133+ cells [111]. Also, these cells significantly inhibited tumor growth in vivo, and the tumor tissue exhibited a higher level of CAR gene copies compared to other groups [111]. In a clinical phase II trial (NCT02541370), the antitumor effects of CAR T-133 cells were assessed in patients with advanced HCC [112]. The results showed disease stability in 14 out of 21 patients [112]. The median progression-free survival of HCC patients treated with CAR T-133 cells was 6.8 months, whereas the overall survival was 12 months [112]. In individuals with previously treated advanced HCC, CAR T-133 cell therapy exhibited promising antitumor activity along with a manageable safety profile [112]. Additionally, early alterations in circulating molecules were identified as potential biomarkers indicative of response to CAR T-133 cells [112].

In recent studies, non-viral approaches have been evaluated for the generation of bispecific CAR T cells [113,114]. The sleeping beauty transposon system from minicircle vectors was used to generate CD133-specific CAR T cells secreting PD-1 blocking scFv (CD133 CAR T and PD-1 cells) [113]. This system demonstrated reduced immunogenicity, lower cost, and significantly increased safety compared to viral vectors [113]. The results also showed high efficacy of the CD133 CAR T and PD-1 scFv cells through in vitro and in vivo experiments, suggesting that a strategy involving CD133 CAR T and PD-1 scFv cells could be a feasible therapeutic option for male patients with advanced HCC and elevated CD133 expression [113]. Non-viral minicircle DNA (mcDNA) vector has also been investigated for the insertion of anti-CD133 and anti-GPC3 scFv structures into T cells [114]. The generation of CAR T cells by this technology yielded exceptional transfection efficacy while, on the other hand, prevented adverse effects associated with viral methods [114]. The bispecific CAR T cells prompted a higher count of effector cells targeting double-positive HCC cells, and displayed antitumor features related to cancer stem cells, thus, effectively disrupting the tumor microenvironment [114]. Moreover, the incorporation of parallel-connected scFv structures on CoG133-CAR T cells facilitated precise recognition, both in vitro and in vivo [114]. Prolonged survival and tumor elimination were observed in Huh7 xenograft mice which received CoG133-CAR T cells, highlighting the substantial potential of mcDNA vectors and bispecific CAR T cells [114].

### 6.4. CD147

CD147 is a type I transmembrane glycoprotein which is highly expressed in most liver cancers and various other malignant tumors [115]. Studies have demonstrated that CD147 plays a pivotal role in promoting tumor progression, invasion, and metastasis through activation of the production of matrix metalloproteinases (MMPs) [116]. Moreover, CD147 expression has been associated with aggressive behavior and poor prognosis in HCC patients [115,117]. A specific monoclonal antibody ^131^I-labeled CD147 has been developed and utilized combined with radiofrequency ablation or TACE for HCC treatment, suggesting its potential use as a targeted therapy for HCC [118]. A novel inducible CAR T cell, regulated by the Tet-On system, was capable of reverse activation or deactivation of the CAR gene expression in the presence or absence of doxycycline (Dox) [119]. Dox can be promptly interrupted if severe adverse events occur, leading to the return of CD147 CAR expression on T cells to baseline within 24–48 h [119]. In in vitro experiments, (Dox+) Tet-CD147CAR T cells exhibited increased cytotoxicity and elevated cytokine production compared to (Dox−) Tet-CD147CAR T cells and peripheral blood mononuclear cells [119]. In parallel, (Dox+) Tet-CD147CART cells effectively inhibited the growth of cancer cells in the HCC xenograft model [119]. A logic-gated (log) GPC3-synNotch-inducible CD147 CAR was also generated to mitigate potential on-target/off-tumor toxicity [120]. LogCD147-CAR specifically targeted and lysed dual-antigen (GPC3+CD147+) without affecting single-antigen (GPC3-CD147+) positive cells [120]. Importantly, no severe toxicity was observed in a human CD147 transgenic mouse model (hCD147TG) [120].

### 6.5. NK Group 2 Member D (NKG2D)

NKG2D is a type II transmembrane glycoprotein that is predominantly expressed on cytotoxic immune cells, NK cells, several autoreactive CD4+ T cells, and subsets of γδ T cells [121]. Typically, NKG2D ligands (NKG2DL) are absent from normal cells but show increased expression on tumor cells, featuring them as potential targets for immunotherapy [122]. The second-generation human NKG2D CAR T cells effectively eradicated NKG2DL-expressing HCC cells, in vitro [122]. However, their efficiency decreased when dealing with NKG2DL-silenced or -negative cells [122]. A subcutaneous xenograft model illustrated that NKG2D CAR T cells significantly inhibited tumor growth [122]. Intriguingly, NKG2D CAR T cells obtained from HCC patients exhibited anti-tumor capabilities and, more specifically, they eliminated HCC cells with high expression of NKG2DL [122]. Non-viral technology was used to prepare NKG2D CAR T cells, utilizing electroporation of CAR-encoding piggyBac transposon plasmids and in vitro expansion with K562 artificial antigen-presenting cells [123]. This approach not only did not compromise the in vitro anti-tumor activity of NKG2D CAR T cells, but also reduced the expression of T cell exhaustion markers [123]. In addition, in order to improve the safety profile of NKG2D CAR T cells, a full-length CD20 elimination transgene was integrated tandemly into the CAR construct via the P2A self-cleaving peptide; this incorporation facilitated antibody-dependent cell-mediated cytotoxicity by autologous NK cells and complement-mediated cytotoxicity [123].

Data from clinical trials that evaluated NKG2D CAR T cells seems promising, and ongoing research focuses on refining CAR constructs and exploring combination therapies [124]. While most trials involve the use of αβT cells, the utilization of NKG2D CAR NK cells or γδ CAR T cells represents an innovative approach that enables autologous cell transfer presenting the potential for off-the-shelf therapies [124].

### 6.6. Epithelial Cell Adhesion Molecule (EpCAM)

EpCAM, a transmembrane glycoprotein, exhibits high expression in numerous human cancers originating from epithelial tissues including HCC [125]. Data has shown that EpCAM(+) HCC cells display hepatic stem cell-like characteristics, such as self-renewal and differentiation, which play pivotal role in the growth and invasiveness of HCC [126]. Additionally, HCC patients with EpCAM+AFP+ demonstrated higher survival and portal vein invasion rates compared to those with EpCAM−AFP− [127], implicating its potency as an early biomarker and therapeutic target for HCC. Currently, EpCAM CAR T cells are being developed for the treatment of colorectal cancer [128], but their application in HCC has not yet been explored. Several clinical trials are under recruitment to evaluate the efficacy and safety of EpCAM CAR T cells in patients with advanced HCC or in cases of postoperative relapse, as well as in refractory HCC (NCT02729493, NCT03013712, NCT05028933).

### 6.7. c-Met

c-Met consists of a tyrosine kinase receptor encoded by the *MET* proto-oncogene that possesses a high-affinity ligand known as hepatocyte growth factor (HGF). Upon binding to HGF, c-Met activates downstream MAPK, STAT3 and PI3K pathways [129], leading to a cascade of biological functions [130]. The HGF/c-Met signaling pathway plays a vital physiological modulatory role in the growth and development of various tissues. Evidence has highlighted the role of c-Met as an activator of hepatocyte proliferation, survival, and regeneration [131]. However, c-Met overexpression can contribute to the development and progression of HCC, limiting this molecule’s potential as a therapeutic target for HCC. Several c-Met inhibitors, such as tivantinib, INC280 and cabozantinib, are currently under investigation in HCC patients [132,133]. The design of bispecific CAR T cells targeting both c-Met and PD-L1 demonstrated significant cytotoxicity against c-Met+PD-L1+ HCC cells [134]. Furthermore, dual-targeted T cells exhibited potent growth suppression activity compared to single-targeted CAR T cells in vivo [134]. Huang et al. developed the second and third generation of c-Met CAR T cells and assessed their anti-tumor efficacy, both in vitro and in vivo [135]. Their findings confirmed the lysis of HCC cells by c-Met CAR T cells, with the third-generation CAR T cells demonstrating enhanced anti-tumor capabilities in vivo [135]. In a recent study, CAR T cells specific to target HCC with MET overexpression were assessed, irrespective of MET activation status. MET-specific CARs CD28ζ and MET-specific CARs 4-1BBζ were constructed; in comparison to MET-CAR.4-1BBζ, MET-CAR.CD28ζ T cells exhibited increased effectiveness against HCC but also displayed a heightened degree of T cell exhaustion [136].

### 6.8. Mucin 1 (MUC1)

Mucin 1 (MUC1) is a transmembrane glycoprotein primarily located to the apical membranes of normal epithelial cells. However, it is overexpressed in various epithelial tumors and some hematological ones. MUC1 serves as an anti-inflammatory molecule; however, prolonged activation can contribute to cancer development including HCC [137]. Reports have shown that the MUC1/JNK/TGF-β signaling pathway promotes the migration and invasion of HCC cells [138]. Therefore, MUC1 is considered an appealing target for HCC therapy. Both first and third-generation CAR T cells targeting MUC1 have been developed, which are able to specifically eliminate HCC cells with high MUC1 expression, while causing minimal damage to normal hepatic cells with low MUC1 expression levels [139]. A recent study has reported the use of Tn-MUC1-targeted CAR T cells in intrahepatic cholangiocarcinoma (ICC) [140]. Tn-MUC1 expression was associated with poor ICC prognosis [140]. Moreover, Tn-MUC1-targeted CAR T cells could selectively eliminate Tn-MUC1-positive ICC cells, both in vitro and in vivo, while there was no effect in Tn-MUC1-negative ICC cells [140]. Currently, a clinical trial involving MUC1 CAR T cells for HCC is actively underway (NCT02587689).

### 6.9. Other Targets

Delta-like homologue 1 (DLK1), a transmembrane protein, exhibits increased expression in HCC [141]. Zhai et al. assessed the potential of DLK1-targeted CAR T cells and the results showed robust cytotoxic activity against DLK1-positive HCC cells in vitro and in vivo [142]. Moreover, the DLK1-directed CARs promoted T cell proliferation and activation in a DLK1-dependent manner. Notably, DLK1-targeted CAR T cells effectively restrained both subcutaneous and peritoneal xenograft tumors originating from human liver cancer cell lines HepG2 or Huh-7 [142].

Elevated serum levels of carcinoembryonic antigen (CEA) have been observed in various adenocarcinomas suggesting a potential use of CEA as a prognostic indicator for HCC [143]. In a phase I clinical trial, six patients with liver metastases underwent hepatic artery infusions (HAI) without experiencing toxicity. Remarkably, one patient remained alive and stable 23 months post-treatment, underlying the safety of CEA CAR T HAI treatment (NCT02416466) [144]. Subsequently, in the phase 1b Hepatic Immunotherapy for Metastases-Selective Internal Radiation (HITM-SIR) trial, six patients with CEA+ liver metastases received anti-CEA CAR T HAI in conjunction with selective internal radiation therapy (SIRT). Importantly, no severe CRS or neurotoxicity occurred during the trial, re-affirming the safety profile of CAR T therapy [145,146].

## 7. Strategies to Improve the Efficacy of CAR T Cell Therapy for HCC

**Figure 4:**
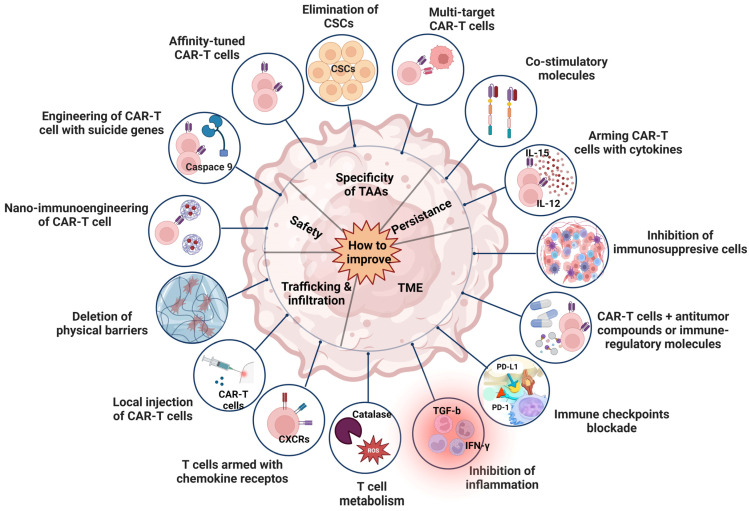
Proposed approaches to ameliorate CAR T cell efficacy against HCC cells. Created with BioRender.com (accessed on 5 January 2024). Abbreviations: CSCs, cancer stem cells; CAR, chimeric antigen receptor; TAA, tumor-associated antigen; IL-15, interleukin 15; TME, tumor microenvironment; IFN-γ, interferon γ; TGF-β, transforming growth factor beta; CXC, chemokine receptor.

Novel approaches and strategies have been utilized aiming to design more powerful CAR T cells with ameliorated anti-tumor activity and decreased toxic effects in order to overcome the significant challenges that have emerged (Figure 4).

### 7.1. Improvement of Persistence

The infusion of CAR T cells into the human body leads to T cell exhaustion. This process limits antitumor effects, primarily due to the blunt response resulting from chronic antigenic stimulation. The cells lose their capacity to proliferate, secrete cytokines and eradicate tumor cells, and express high levels of inhibitory receptors including PD-1, TIM-3 and LAG-3 [147]. In order to enhance their persistence, CAR T cells expressing various costimulatory molecules were constructed [148]. Additional strategies involve augmentation of the expression of cytokines in the CAR structure, such as IL-12, IL-15 and IL-21, in order to promote CAR T cell proliferation and to increase T naïve cell expression [149].

### 7.2. Improvement of TME

Although CAR T cells can reach tumor sites successfully, their ability to exhibit anti-tumor effects is hindered. Only a limited number of cells demonstrate efficacy due to the prevailing immunosuppressive microenvironment. This environment is characterized by various inhibitory factors, including metabolic elements, inflammatory factors, immunosuppressive cells, and immune checkpoints. Thus, consideration should be given to gene editing against immune checkpoints on TAA CAR T cells, coupled with targeted drugs to negate the tumor’s immunosuppressive microenvironment and to improve the local metabolism [150,151,152]. This intricate network collectively diminishes HCC growth through inhibition of T cell attacks.

### 7.3. Improvement of Trafficking and Infiltration

Typically, CAR T cells are administered via peripheral infusion, and their ability to migrate to the tumor site is imperative for the achievement of cytolytic effects. However, T cells typically lack the expression of chemokine receptors, which are instrumental in T cell transportation to tumor sites through their binding to chemokines produced by tumor cells [153]. Furthermore, HCC tissue creates a dense fibrotic matrix, leading to the downregulation of chemokine expression. This substantially diminishes the migration and infiltration capabilities of CAR T cells towards the tumor. To enhance the trafficking and infiltration abilities, chemokine receptors bearing CAR T cells [154] and CAR T cells expressing heparinase have been developed [155]. In parallel, endeavors for local injection of CAR T cells significantly improved the antitumor effects [156].

### 7.4. Improvement of Safety

CAR T cell infusion often results in significant adverse effects, including on-target, off-tumor toxicity, CRS and neurotoxicity among others. Hence, enhancing the safety of CAR T cells is of paramount importance. Introduction of novel systems, such as the Tet-On inducible gene system can effectively regulate the expression and activity of CAR [119]. In case of CRS which leads to neurotoxicity, CAR expression can be promptly deactivated, increasing their safety compared to the traditional CAR T cells. For example, incorporation of the *iCaspase-9* gene into the structure of CARs could facilitate the disruption of T cell activation by inducing apoptosis in CAR T cells. The use of inhibitors for extracellular vesicles (EVs), which function as carriers transporting functional molecules that promote tumor growth and metastasis, potentially interfering with CAR immunotherapy, is a viable approach [157]. Preliminary data have shown that nanomedicine has great potential for treating HCC [158,159]. Photothermal therapy is an innovative strategy to improve the accumulation and function of CAR T cells within the solid tumors by using light-absorbing substances to generate heat when exposed to light, typically in the near-infrared (NIR) range [160]. Lastly, structural modifications have demonstrated the potential to reduce CAR T cells toxicity while maintaining their efficacy in tumor elimination [161]

### 7.5. Improvement of Specificity

One major challenge impeding the success of CAR T cell therapy against HCC lies on the difficulty of pinpointing the ideal TAAs. In numerous solid tumors, only a subset of cells expresses the target antigen. Even if the TAAs are consistently expressed in the HCC tissue, there still exists a risk for antigen loss or antigen escape, contributing to notable antigen heterogeneity. Due to this heterogeneity, a singular CAR T therapy approach may not be effective for all HCC patients. Thus, a promising strategy involves targeting multiple TAAs. Additionally, TAAs are not exclusive to cancer cells; they are also expressed at low levels in normal cells, leading to on-target, off-tumor toxicities in healthy tissues. Thus, dual-targeted CAR T cells [162,163], affinity-tuned CAR T cells [164], and CAR T cells targeting cancer stem cells [111] were constructed to ameliorate the specificity of tumor antigens.

### 7.6. Improvement of Drug Resistance

Drug resistance during CAR T cell therapy refers to cancer cells’ development of mechanisms to evade or counteract the effects of CAR T cells, leading to a reduction in treatment efficacy [165]. Addressing and overcoming this challenge in CAR T cell therapy is complex. Cancer cells may downregulate or completely lose the expression of the targeted antigen, making them less susceptible to CAR T cell recognition and, subsequently, resistant to treatment [166]. In addition, tumor cells may exhibit heterogeneity in antigen expression, as cancer cells within the same tumor may lack the targeted antigen, thus escaping CAR T cell detection. To overcome these challenges, researchers are actively exploring various strategies to enhance the effectiveness of immunotherapies. Such strategies could be the use of combination therapies; checkpoint inhibitors in conjunction with CAR T cell therapy can enhance T cell activity and overcome immune suppression. Moreover, combination of different immunotherapies, such as CAR T cell therapy with oncolytic viruses or cancer vaccines, may lead to synergistic effects. Moreover, one further step to overcome drug resistance could be the modulation of the cytokine milieu within the TME; this might help to create a more supportive immune context for CAR T cell function. Engineering CAR T cells with the ability to recognize multiple antigens simultaneously can broaden their target specificity, reducing the risk for antigen loss escape. Alongside, the incorporation of a third specificity in CAR T cells can further enhance their ability to recognize and attack cancer cells. Genetic engineering may be one more step towards improvement of CAR T cells persistence; cytokine secretion or resistance to inhibitory signals may enhance CAR T cells’ therapeutic effects or the blockade of immune checkpoint inhibition can help maintain CAR T cells’ functionality in the presence of inhibitory signals. Lastly, further optimization of CAR framework structures and new CARs design, such as the arrival of switchable CARs, logic-gated CARs, or armored CARs, may provide greater control and flexibility in response to evolving challenges, particularly in solid tumors which present unique features due to the biological complexity of the solid TME.

## 8. Conclusions and Prospects

The introduction of CAR T cells has revolutionized the treatment landscape for specific hematological malignancies, extending CAR T cell application to solid tumors. However, despite these advancements, numerous challenges persist. The selection of antigens holds paramount importance in the functionality of CAR T cells. In the context of HCC, the direction of CAR T cells towards tumor infiltration poses a great challenge, especially when considering the immunosuppressive microenvironment. Additionally, effective treatment introduces the risk for CAR T cell-associated toxicities, such as CRS and neurotoxicity. Despite these challenges, ongoing research offers a promising path towards more efficacious and safer future therapies. Over recent years, in depth understanding of the immunosuppressive environment has led to novel approaches augmenting tumor elimination and sustained cytotoxicity of CAR T cell therapy. Several clinical trials of CAR therapy in HCC are underway to confirm improved outcomes. Administration of antitumor-associated cytokines may improve immune responses and intensify the HCC cell-killing effect of CAR -T cells. Combined therapies integrating enhanced T cell activity (such as infiltration and precise targeting) with improvements in the intra-tumoral environment (such as addressing physical and biochemical barriers), may pose effective approaches for HCC treatment. Assessing photothermal therapy or the blockade of EVs released in the HCC TME could prove crucial to overcome the challenges presented with TME. Additionally, understanding the molecular mechanisms within the TME enables researchers to adeptly design molecular targets for CAR cells in order to restore sensitivity to TME resistance.

Although CAR T cell therapy has shown remarkable outcomes, there remains a long way ahead in CAR research to establish an applicable treatment for solid tumors, particularly in the setting of HCC. Future research should focus on a more in-depth exploration of the mechanisms governing the establishment of tumor barriers and the intricate structural characteristics involved. This exploration has the potential to reveal an extended range of therapeutic targets suitable for sequential interventions. Undoubtedly, the convergence of various treatment modalities, coupled with the continuous progress in scientific investigation, holds promise to advanced HCC patients’ care.
